# Computational Screening of the Natural Product Osthole and Its Derivates for Anti-Inflammatory Activity

**DOI:** 10.3390/life12040505

**Published:** 2022-03-30

**Authors:** Angela Mosebarger, Rambabu N. Reddi, Ramkumar Menon, Ananth Kumar Kammala

**Affiliations:** 1 Division of Basic & Translational Research, Department of Obstetrics & Gynecology, The University of Texas Medical Branch at Galveston, 301 University Blvd., Galveston, TX 77555, USA; anmoseba@utmb.edu (A.M.); ra2menon@utmb.edu (R.M.); 2 Department of Organic Chemistry, Weizmann Institute of Science, Rehovot, 234 Herzl St., P.O. Box 26, Rehovot 7610001, Israel; rambabu.reddi@weizmann.ac.il

**Keywords:** osthole, molecular docking, semi-synthetic derivatives

## Abstract

Osthole (OS) is a natural coumarin with a long history of medicinal use in a variety of diseases, such as itch and menstrual disorders. In recent years, OS has been shown to treat inflammation and reduce the expression and activity of NF-κB, although its mechanism of action is still unclear. Overexpression of inflammatory cytokines can have many negative effects in the body, including inducing preterm labor; thus, the modulation of inflammation by OS and its derivatives may be able to delay preterm birth, increasing neonatal survival rates. The objectives of this study were to screen and identify the derivatives of OS with the highest potential for binding capacity to inflammatory mediators NF-κB, TNF-α, and ERK1, and to measure the drug-like properties of these compounds. GLIDE docking in Schrodinger Maestro software was used to calculate docking scores for a variety of semi-synthetic OS derivatives against three proteins involved in inflammation: NF-κB, TNF-α, and ERK1. Schrodinger Qikprop was also used to measure the pharmaceutically relevant properties of the compounds. The protonated demethoxy osthole **1** showed the highest docking of all the proteins tested, while the deprotonated demethoxy osthole **2** consistently had the lowest scores, denoting the importance of pH in the binding activity of this derivative. The lowest docking was at NF-κB, suggesting that this is less likely to be the primary target of OS. All of the screened derivatives showed high drug potential, based on their Qikprop properties. OS and its derivatives showed potential to bind to multiple proteins that regulate the inflammatory response and are prospective candidates for delaying preterm birth.

## 1. Introduction

Recent advancements in drug discovery have increased attention towards natural compound-based pharmacotherapy for various disorders [1,2,3]. Novel computational methods have opened up new possibilities for processing complex natural products and using their derivates to develop novel drugs [4]. Traditional Chinese medicine (TCM) and the Indian system of medicine (ISM) have been using the plants called *Cnidium monnieri (L) Cusson* [5,6], and *Angelica archangelica Linn* [7,8,9], respectively, for various disease conditions, such as itchy skin [10], eczema [7], erectile dysfunction [4,6,11], cancer [12], and osteoporosis [13,14,15,16], as well as fungal and bacterial infections [17,18]. Osthole (7-methoxy-8-(3-methyl-2-butenyl)-2H-1-benzopyran-2-one) is a natural coumarin and a major chemical constituent in the above-mentioned plants [15,17,18,19], and has been recognized as a promising lead compound in drug discovery research associated with various pharmacological activities, such as anti-cancer [19,20], anti-inflammatory [21,22,23], antioxidative [20,24], anti-angiogenic activity [25], antiallergic [26,27,28], immunomodulation [27,29,30], and hepatoprotective activities [31,32,33,34,35]. The molecular mechanisms of the osthole (OS) pharmacological actions involve the inhibition of signaling molecules such as nuclear factor kappa B (NF-κB) [36,37], tumor necrosis factor alpha (TNF-α) [38,39], and mitogen-activated protein kinases (MAPK) [40,41,42] that are involved in various processes of the immune response and metabolic and biological processes. Since OS has a wide variety of pharmacological applications and is well established as an inhibitor of the phosphorylation of inflammatory transcription activator NF-κB, TNF-α, and MAPK-related proteins [22,23,27,30,43,44,45], it could also be a potential therapeutic molecule for various pregnancy-related disorders, such as spontaneous preterm birth and preeclampsia, where NF-κB [46,47,48,49,50], TNF-α, and MAPK-related proteins play a major role. Based on recent studies, the inflammatory response to infection is believed to be the causative agent in the premature induction of labor. Infection-induced inflammation increases cytokines and apoptosis, leading to the preterm premature rupture of fetal membranes [51,52].

Although OS has several pharmacological activities, it has extremely low bioavailability in rat plasma pharmacokinetics [53,54]. Considering the drug-like properties of OS, it has a low molecular weight and high cLogP, but lacks a hydrogen-bond acceptor and exhibits low water solubility and bioavailability. In order to enhance its drug-like properties, a more polar group, such as a hetero atom or heterocyclic aryl ring, could be considered for introduction to the lead molecule, to improve its bioactivity and physicochemical properties [31,55]. The unique structural features of OS exhibit comprehensive biological parameters and make it a good candidate for the generation of lead molecules. Therefore, in the present study, we have explored the possibility of synthesizing various OS derivatives, mainly by modifying at the seventh position and the fourth position. Before producing these semi-synthetic derivatives of OS, they were screened to dock with signaling molecules such as NF-κB, TNF-α, and MAPK-related proteins. The goal of this research was to analyze the potential of drug molecules to inhibit the inflammatory process via binding to these proteins, and their capability of crossing physiological barriers without any toxic effects, in order to identify candidates for the treatment of inflammation in conditions such as preterm birth. 

## 2. Materials and Methods

### 2.1. Designing of OS Derivatives

**Figure 1:**
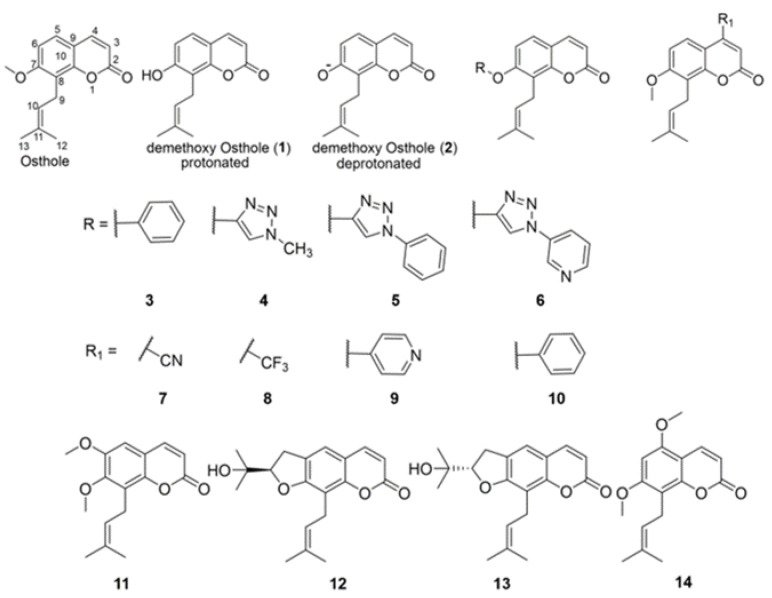
Chemical modification of osthole (OS) and scheme for synthesizing OS derivatives. At the 7th and 4th positions of the OS, chemical modifications were proposed to improve their pharmacological activity and bioavailability. The modifications at 7th position include adding phenolic group (**3**), aromatic and heteroaromatic triazole motifs (**4**–**6**). The modifications at 4th positions are electron-withdrawing cyano (**7**), trifluoro (**8**), and aromatic and heteroaromatic functionalities. The other modifications include at 6th and 5th position (**11** and **14**), and a chiral cyclic ring structure at 6–7th position (**12** and **13**).

We have designed several OS derivatives, modifying at the 4th and 7th positions (Figure 1). All the proposed modified OS derivatives are either synthetically possible or commercially available. OS has a methoxy (-OMe) group on the 7th position. We modified this group by removing the methyl (protonated: **1**, deprotonated: **2**) and also modified the 7th position with a phenoxy (**3**) group and with three triazoles containing aromatic and heteroaromatic methoxy groups (**4**–**6**). Furthermore, we modified the OS compound at the 4th position, adding various electron-withdrawing groups, such as -CN (**7**) and -CF3 (**8**), and heteroaromatic and aromatic rings (**9** and **10**). In addition to these derivatives, we performed double modifications at the 6th and 5th positions using methoxy groups (**11** and **14**) and also used a chiral cyclic ring structure at the 6–7th position (**12** and **13**). These semi-synthetic derivative compound designs were subjected to Schrodinger Maestro software, to predict their physicochemical properties and pharmacokinetic parameters.

### 2.2. Structures of Anti-Inflammatory Mediators

NF-κB (PDB:1SVC) has been crystallized bound to DNA [56]. The crystal structure in the Protein Data Bank (PDB) is not bound to a small molecule, so dehydroxymethyl-epoxyquinomicin (DHMEQ), a known NF-κB inhibitor, was used as a binding control instead [57]. Two potential binding sites were detected on this structure, both located in the DNA binding region (Tables S1 and S2). TNF-α (PDB: 2AZ5) was co-crystallized with an inhibitor [58], the binding of this inhibitor forming the TNF-α dimer. 

Two different crystal structures were used to study ERK1 (PDB: 4QTB [59] and 2ZOQ [60]). In the structure 2ZOQ, ERK1 is monophosphorylated at Tyr 204, allowing basal level activity, and is complexed with a potent inhibitor, 5-iodotubericidin (5-IOD). The residues around this binding site are conserved between ERK1 and ERK2, and the bound inhibitor is active at both isoforms. This protein has other binding sites that may be isoform-specific, but they were not tested here. The other crystal structure of ERK1 (4QTB) is bound to SCH772984, a selective inhibitor of both ERK1 and ERK2. This inhibitor creates a novel binding pocket, distorting the protein to block ATP binding and forming an inactive state. Chaikuad et al. identified water-mediated interactions at Q117, K511, and Q105 in ERK1 and ERK2 bound to SCH772984 (numbering based on ERK2) [59,60]. Unlike the other structures, five water molecules within 5Å of the docked ligand failed to show hydrogen bonding to the protein or ligand in 4QTB, so this protein was screened in three conditions (water removed, water included in the binding site, and limited water, where water molecules that do not show hydrogen bonding interactions are removed), while the other structures were only screened with all water removed and all water included within 5Å of the docked ligand, because all of the water molecules present in these structures showed hydrogen bonding to the protein or ligand (Table S3). The data shown in the present study represent the proteins including only water molecules that demonstrate hydrogen bonding in the binding pocket, except in the case of the modified 1SVC, which is shown with all water molecules removed. 

### 2.3. In Silico Docking of Co-Crystallized Proteins

Docking was performed using GLIDE in Maestro 12.9. Four proteins were imported from the PDB: NF-κB (1SVC), TNF-α (2AZ5), and ERK1 (2ZOQ and 4QTB). TNF-α and ERK1 had small molecules bound to the protein in their crystal structures, so these ligands were used as positive controls in binding studies. However, NF-κB was bound to DNA rather than a small molecule; because of this, binding sites on the NF-κB protein were identified with Maestro binding site detection software, and DHMEQ, a known inhibitor of NF-κB, was used as the control ligand [57]. The proteins co-crystallized with ligands were imported from the PDB and separated from extra entities (i.e., SO4 ions in ERK1). Using Maestro’s Protein Preparation Wizard, the proteins were assigned bond orders, hydrogens were added, disulfide bonds were created, and waters beyond 5Å from the bound ligand were deleted. Missing atoms were filled in using Prime. After preprocessing, the protein was checked for alternate positions of residues. The position that allowed the most favorable interactions was committed. If neither affected docking, the highest average occupancy was chosen. Next, the protein was refined with H-bond assignment and a restrained minimization at RMSD of 0.30Å and OPLS4 forcefield. All protein and ligand processing was undertaken at a fetal physiological pH of 7.3 ± 2. In cases of homodimers, only one chain was used, to simplify processing. The ligands were generated using the NCI Online SMILES Translator and Structure File Generator and imported as mol files. They were prepared using LigPrep in Maestro at pH 7.3 ± 2, and all stereoisomer combinations were generated. Ligands were also aligned using the prepared control as a reference and the largest common Bemis–Murcko scaffold as the conformation. The protein was duplicated to create an entry that included the waters at the binding site, an entry with no waters, and an entry that only included waters that showed hydrogen bond interactions with the protein or ligand (only 4QTB contained waters that did not have those interactions). A receptor grid was generated for each version of the protein using the unprepared ligand from the crystal structure, and the prepared ligands were docked onto that grid using Grid-based Ligand Docking with Energetics (GLIDE 6.0) [61,62]. Ligands were docked with extra precision, and Epik state penalties were added to the docking score. Images were saved using the ligand interaction tool. The Qikprop tool in Maestro was used to calculate drug-like properties for each of the ligands, after they were processed with LigPrep [63]. The locations of each binding site are visible in Figure S1 and the active residues are shown in Table S4.

### 2.4. In Silico Docking of 1SVC (NF-κB)

NF-κB was imported to Maestro using its PDB 1SVC, and all waters were initially removed. Because this crystal structure has a mutation at residue 62, Maestro software was used to modify the alanine 62 to a cysteine, creating the wild-type sequence. Both versions were analyzed in the present study. Using Maestro’s Protein Preparation Wizard, the protein was assigned bond orders, hydrogens were added, and disulfide bonds were created. The protein was then refined with H-bond assignment and a restrained minimization at RMSD of 0.30Å and OPLS4 forcefield. The protein was also processed at a fetal physiological pH of 7.3 ± 2. The 3D structure of DHMEQ was downloaded from PubChem and converted to a mol file via the NCI Online SMILES Translator and Structure File Generator, before importing to Maestro. The ligand was processed with LigPrep [63] software in Maestro at fetal physiological pH 7.3 ± 2, and chiralities were determined from the 3D structure. The binding site detection tool in Maestro was used to identify potential binding sites on the unmodified protein (Table S1), while the cysteine 62 was used as the docking site for the modified 1SVC, and DHMEQ was docked at those sites to maximize positive interactions between the ligand and the protein. The waters were then added back to the protein, and it was processed again in the same way, except, this time, the Protein Prep Wizard was also used to delete any waters beyond 5Å from the docked ligand. The rest of the ligands to be screened were also generated using the NCI Online SMILES Translator and Structure File Generator and imported into Maestro. They were prepared using LigPrep at pH 7.3 ± 2, and all stereoisomer combinations were generated. Ligands were also aligned using the prepared control as a reference and the largest common Bemis–Murcko scaffold as the conformation. The protein was duplicated to create an entry that included the waters at the binding site and an entry with no waters. A receptor grid was generated for each version of the protein using the docked DHMEQ, and the prepared ligands were docked onto that grid using GLIDE. Ligands were docked with extra precision, and Epik state penalties were added to the docking score. 

### 2.5. Analysis of Docking Scores

The conformational and ionic state of the co-crystallized ligand with the best docking score was used as the control to compare to screened derivatives. Site 2 of the unmodified NF-κB showed very low docking scores for its positive control DHMEQ (Table S5). The sites for NF-κB were generated by binding site detection on Maestro, not via known binding sites or co-crystallization. While DHMEQ is a known inhibitor of NF-κB, it is likely that it does not bind to site 2, and there is no evidence that binding here could inhibit the action of the protein. For this reason, site 1 of NF-κB was analyzed further, but site 2 was excluded. The modified 1SVC had higher scores and was also analyzed further. The structure 4QTB also showed low scores for controls relative to the screened ligands. In this case, the binding site is known to bind to the inhibitor, because it comes from co-crystallization. The docking results could be due to the fact that the derivatives screened are such good binders that they score better than the bound control. Therefore, this structure was analyzed further.

## 3. Results

### 3.1. NF-κB Binding Site

**Figure 2:**
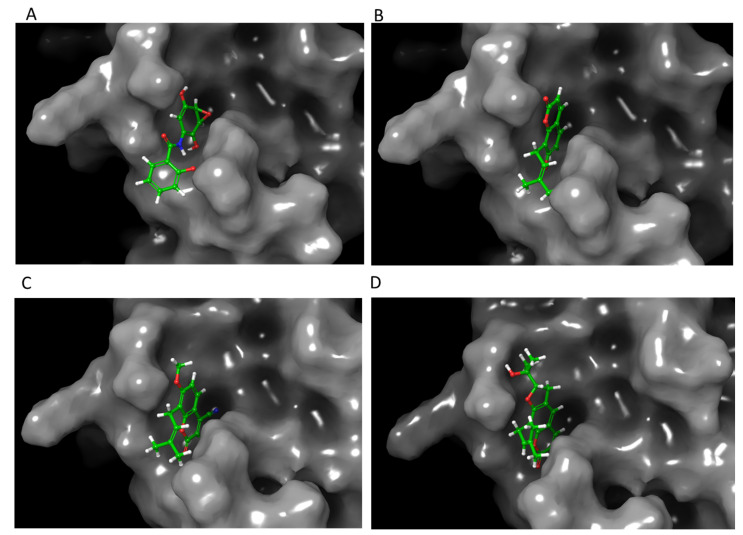
Molecular modeling of top-scoring compounds docked at Cys62 in modified 1SVC (NF-κB) with no water included in binding site. (**A**) Known inhibitor DHMEQ. (**B**) Ligand **1**. (**C**) Ligand **7**. (**D**) Ligand **13**.

**Figure 3:**
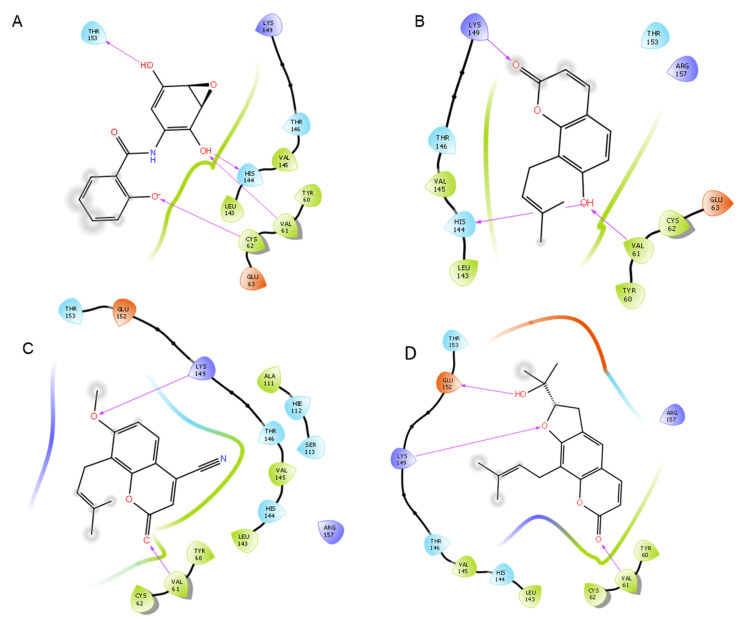
Protein–ligand interactions of top-scoring compounds docked to modified 1SVC (NF-κB), water molecules not included in binding site. (**A**) Known NF-κB inhibitor DHMEQ. (**B**) Ligand **1**. (**C**) Ligand **7**. (**D**) Ligand **13**.

NF-κB was analyzed using the structure on the Protein Data Bank 1SVC. This is the human p50 subunit of NF-κB bound to DNA. Because this structure was crystallized bound to DNA rather than a small molecule, there was no known inhibitory binding site in the crystal structure. NF-κB inhibition can result from a variety of mechanisms that may or may not include direct binding to NF-κB [64]. For the purpose of this study, the mechanism analyzed was that of the known inhibitor DHMEQ, which works by binding to cysteine 62 of p50 [64]. However, the sequence given for 1SVC on the Protein Data Bank includes a mutation, containing an alanine at residue 62 instead of cysteine to improve crystallization, although the other residues are unchanged [65]. This residue was modified in silico to cysteine using Maestro software in order to test the wild-type protein with a cysteine at residue 62. Docking simulations were run both on the mutated protein (1SVC; NF-κB C62A) using binding site detection software (Figures S2 and S3), as well as the wild type (modified 1SVC; NF-κB) using the cysteine 62 residue as the basis for docking (Figure 2 and Figure 3). The modified 1SVC showed an improvement in docking scores (Table 1).

The sites identified by binding site detection software did not include residue 62 (binding site scores and residues shown in Tables S1 and S2), and the top-scoring ligands did not show major interactions with this residue (Table 2). However, in the modified version, when cysteine 62 was used for docking, the scores showed a marked improvement in binding, although the three top-scoring compounds (besides DHMEQ) did not show a direct interaction with the cysteine. The modified 1SVC (NF-κB) showed an improvement in docking scores with both DHMEQ as well as the OS derivatives when water was removed from the binding site, while the other structures showed generally better scores with water included (Table S5). This structure was further analyzed without water molecules, while the other proteins were analyzed with water molecules included.

### 3.2. Role of Water in Docking

**Figure 4:**
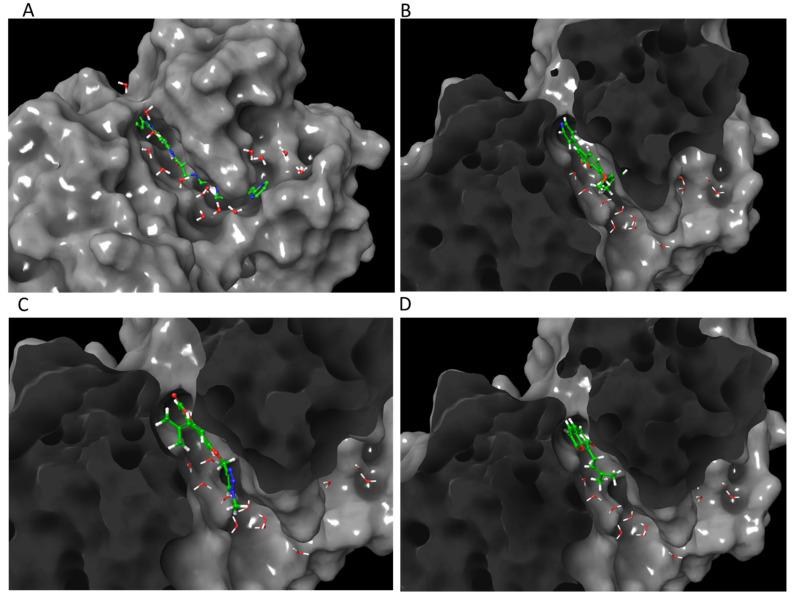
Molecular modeling of top-scoring compounds docked at 4QTB (ERK1) with water included in binding site. (**A**) Known inhibitor SCH772984. (**B**) Ligand **9**. (**C**) Ligand **4**. (**D**) Ligand **1**.

**Figure 5:**
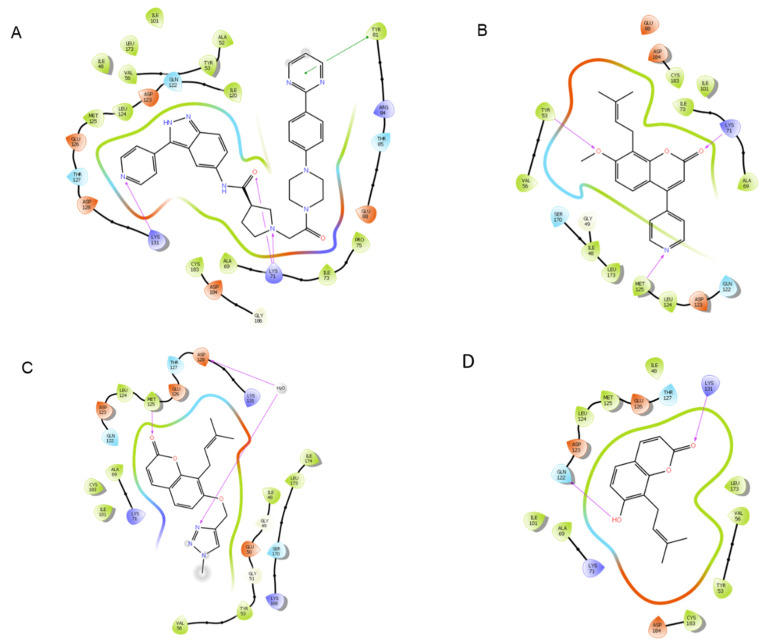
Protein–ligand interactions of top-scoring compounds docked to 4QTB, water molecules included in binding site. (**A**) SCH772984. (**B**) Ligand **9**. (**C**) Ligand **4**. (**D**) Ligand **1**.

**Figure 6:**
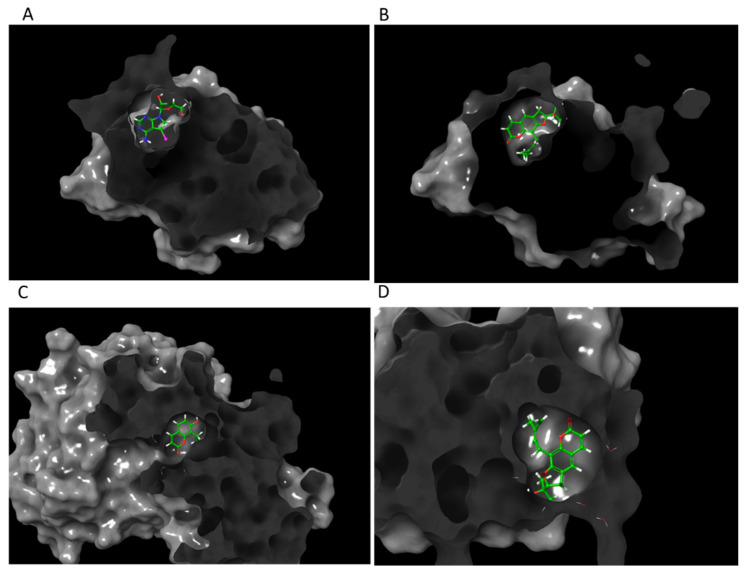
Molecular modeling of top-scoring compounds docked at 2ZOQ (ERK1) with water included in binding site. (**A**) Known inhibitor (2*r*,3*r*,4*s*,5*r*)-2-(4-amino-5-iodo-7h-pyrrolo[2,3-d]pyrimidin-7-yl)-5-(hydroxymethyl)tetrahydrofuran-3,4-diol. (**B**) Ligand **13**. (**C**) Ligand **1**. (**D**) Ligand **12**.

**Figure 7:**
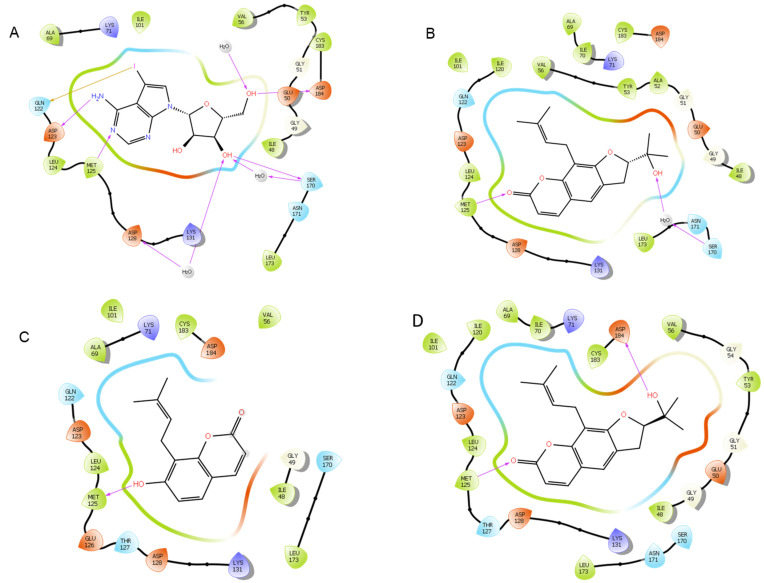
Protein–ligand interactions of top-scoring compounds docked to 2ZOQ, water molecules included in binding site. (**A**) Inhibitor (2*r*,3*r*,4*s*,5*r*)-2-(4-amino-5-iodo-7h-pyrrolo [2,3-d]pyrimidin-7-yl)-5-(hydroxymethyl)tetrahydrofuran-3,4-diol. (**B**) Ligand **13**. (**C**) Ligand **1**. (**D**) Ligand **12**.

When comparing docking scores between water conditions, OS and its derivatives score better when water is present, except on the structure 4QTB (ERK1) and the modified 1SVC (NF-κB). This is likely because the waters are positioned to match the control ligand, so the fit will not work as well for differently shaped compounds. The structure 4QTB even failed to dock OS derivatives **3**, **9**, **10**, and **11** when all of the water molecules were present, but the scores improved when only waters with interactions to the receptor or ligand were included. Modified 1SVC (NF-κB) demonstrated a marked improvement when the waters were removed. As shown in Table 1, the parent compound OS exhibited the best docking score at 2ZOQ (ERK1) with water present, suggesting that ERK1 is the most likely target for OS out of the proteins screened here. It also shows that OS may rely on water interactions similar to the known inhibitor docked in the crystal structure. The binding of inhibitor SCH772984 to ERK1/2 has been shown to form a network of water-mediated hydrogen bonds across the ATP binding site [59]. This can be visualized in Figure 4, where water molecules are seen surrounding the ligand in the binding site. Specific water molecule interactions with 4QTB can be visualized in Figure 5. This is also demonstrated with the other form of ERK1, 2ZOQ, in Figure 6 and Figure 7, where water molecules mediate interactions with specific top-scoring ligands. All interactions with water molecules are shown in Table S3, again demonstrating the high number of water interactions on the 4QTB structure. 

### 3.3. Interactions within Binding Sites

**Figure 8:**
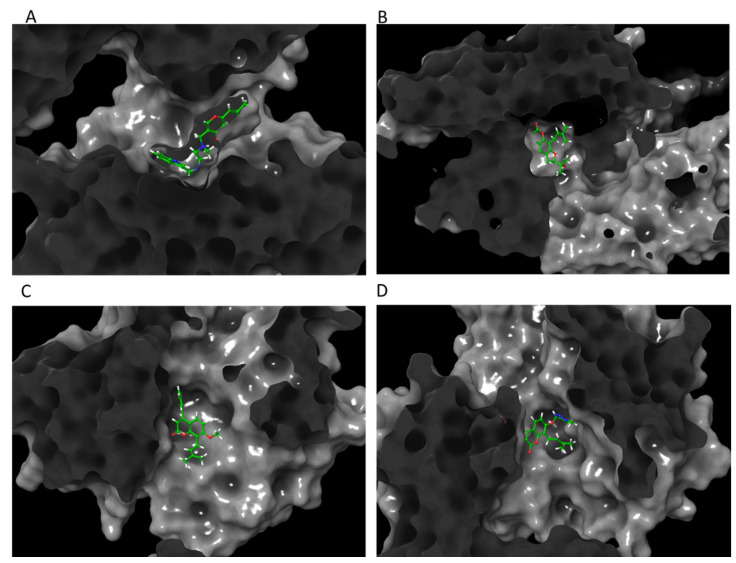
Molecular modeling of top-scoring compounds docked at 2AZ5 (TNF-α) with water included in binding site. (**A**) Known inhibitor 6,7-dimethyl-3-[(methyl{2-[methyl({1-[3-(trifluoromethyl)phenyl]-1h-indol-3-yl}methyl)amino]ethyl}amino)methyl]-4h-chromen-4-one. (**B**) Ligand **12**. (**C**) Ligand **10**. (**D**) Ligand **4**.

**Figure 9:**
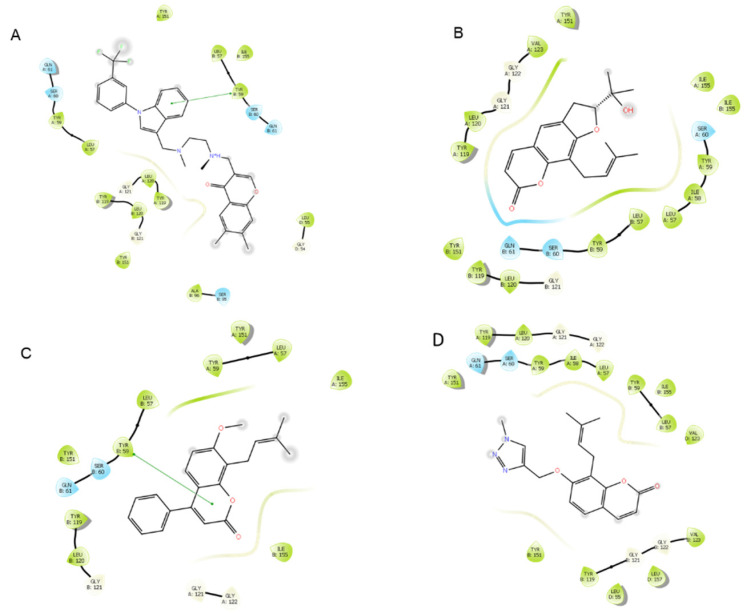
Protein–ligand interactions of top-scoring compounds docked to 2AZ5, water molecules included in binding site. The green lines in diagrams (**A**) and (**C**) represent π-π stacking interactions. (**A**) 6,7-dimethyl-3-[(methyl{2-[methyl({1-[3-(trifluoromethyl)phenyl]-1h-indol-3-yl}methyl)amino]ethyl}amino)methyl]-4h-chromen-4-one. (**B**) Ligand **12**. (**C**) Ligand **10**. (**D**) Ligand **4**.

Active residues in binding sites were identified by showing interactions between the receptor and ligand in the crystallized structure. In the case in which there is not a ligand in the crystal structure (1SVC), the residues were identified by binding site detection software, or by docking DHMEQ at its known target, cysteine 62. Major interactions with the three highest-scoring ligands can be found in Table 2, and total interactions at each binding site can be found in Table S4. In this study, 1SVC (NF-κB C62A) showed no interactions with residue 62 when it was an alanine, but when alanine was replaced with cysteine in the modified 1SVC (NF-κB), it demonstrated a hydrogen bond to DHMEQ. The receptor also demonstrated important hydrogen bond interactions with valine 61 and lysine 149 (Table 2). When the cysteine 62 residue was not present, the most important interactions were a π-cation interaction with lysine 80 and hydrogen bond with glycine 68. Moreover, 4QTB and 2ZOQ (both representing human ERK1) consistently showed interactions with methionine 125, with 4QTB showing more direct interactions than 2ZOQ or any other structure analyzed in the present study. In addition, 2AZ5 (TNF-α) showed π-π stacking interactions and hydrogen bonds with the ligand at tyrosine 59, and other interactions were found at serine 60, leucine 120, glycine 121, and tyrosine 151 (Figure 8 and Figure 9).

### 3.4. Identification of Hits

When using GLIDE software to screen ligands, it is important to remember that docking scores are not the same as binding affinities, as they are only a prediction of goodness of fit in a binding site. A highly negative docking score does not guarantee strong binding, but it increases the probability that there will be binding at that site. Because of this limitation, it is often best to look at leads that show promising docking scores across multiple proteins and sites, rather than only considering those that have a very good docking score at just one site. These derivatives are being screened for general anti-inflammatory properties, meaning that protein selectivity between these anti-inflammatory mediators is not very important, and it is best to begin by considering each compound’s average score across the structures studied. When considering each compound’s average score, **1** was the derivative with the greatest average docking score across the structures screened, while **2** was the derivative with the lowest average docking score. This huge difference between the similar structures suggests the importance of pH for docking to these structures. The highest-scoring protein was 2ZOQ, and the lowest average score was 1SVC, even when the scores were improved by modifying residue 62 and removing water molecules (Figures S4–S6). Hits were also identified for each structure by finding derivatives with the highest docking scores and those that scored better than the parent compound OS. Of these leads, the highest-scoring and most consistent hits were identified as **1**, **4**, **13**, and **14**.

### 3.5. Qikprop

The Qikprop tool from Schrodinger can calculate drug-like properties for screened compounds. From these properties, specific differences can be identified between the compounds, as shown in Table 3. For example, **7**, which contains a cyanide group, is the only compound with a negative logkHSA, meaning that it has lower binding to serum proteins than the other derivatives. There were no deviations of the rule of five (molecular weight < 500, predicted octanol/water partition coefficient logPo/w < 5, hydrogen bond donors < 5, hydrogen bond acceptors < 10) on the screened ligands. Only **5** and **6** had deviations in the rule of three (molecular weight < 300, ClogP < 3, hydrogen bond donors and acceptors < 3). In most cases, all of the compounds fall within the recommended range for drug leads, except for the hERG channel blockage. Around half of the compounds scored a value less than −5, including two of the leads identified here, **4** and **14**, which indicates a risk of cardiac issues associated with the drug. All of the screened derivatives have more negative logBB (less permeability to the blood–brain barrier) than OS, except **8**, which actually exhibits a positive logBB, meaning that it has more CNS-targeting properties than all of the other screened ligands. All of the derivatives have a comparable oral absorption and ionization potential to OS.

## 4. Discussion

The medicinal properties of the natural product OS have been known for centuries but were not tested until recently. It has been shown to decrease neurogenic pain and reduce the generation of reactive oxygen species, as well as preventing DNA fragmentation and damage to mitochondrial membranes. Its therapeutic effects range from cytoprotection and anti-apoptosis to the prevention of myocardial fibrosis. It acts by mediating inflammatory cytokines. Pretreatment with OS has been shown to reduce the expression and activity of the transcription factor NF-κB, as well as other inflammatory signals, such as TNF-α, nitric oxide, and IL-6. It also plays a role in differentially regulating the phosphorylation of p38, JNK1/2, and ERK1/2. Because of its effects on inflammatory cytokines, OS is believed to have the potential to delay preterm labor, which can be induced by inflammation.

This study outlines the protocol of docking ligands to proteins with or without a known binding site and inhibitor. Binding on 1SVC (NF-κB) was decreased when cysteine was replaced with alanine at residue 62. This is consistent with previous findings, and further highlights the importance of this single residue in the inhibition of NF-κB by DHMEQ and possibly other molecules [65]. The binding of OS with ERK1 (PDB: 2ZOQ, 4QTB) also suggests that the binding of OS with this protein may rely on water molecules in the binding site, although the presence of too many water molecules may block the binding of some compounds (PDB: 4QTB).

When analyzing scores calculated by GLIDE software, it is important to understand that docking scores are not equivalent to binding affinity. Due to the rigid structure of the receptors in the program, these calculations are only estimates. To overcome this limitation, it can be helpful to test against multiple structures and identify potential leads that could bind to multiple targets. Because this study was focused on multiple causes of inflammation, and specificity between the studied proteins is not vital, this strategy was utilized in the analysis of these leads. These ligands are being analyzed for their potential as general anti-inflammatory leads, but more specific hits can also be identified to target one protein (i.e., **9** shows good binding at 4QTB, but not to NF-κB or TNF-α, so it may selectively bind to the novel pocket on ERK1/2, identified by Chaikaud et al. [59]) or for more specific actions (**8** shows higher blood–brain barrier permeability than all the other derivatives). The change in effect from **1** and **2** also indicates the importance of pH in the activity of this compound, which may be used for compartment- or tissue-specific targeting. The Qikprop analysis showed that all the screened derivatives have good pharmaceutical properties and have the potential to be used as drugs [65].

## Figures and Tables

**Table 1:** Docking scores (kcal/mol) for each compound with each protein. More negative binding scores correlate to better binding. The binding sites include water molecules that demonstrated hydrogen bonding to the ligand or receptor, but in the case of the modified 1SVC, all water molecules were removed.

	1SVC Site 1	Modified 1SVC	4QTB	2ZOQ	2AZ5	Mean
control	−4.561	−6.46	−3.151	−10.975	−6.166	−6.286
OS	−2.837	−4.294	−4.627	−6.49	−5.057	−4.52625
**1**	−3.381	−5.102	−5.3	−7.375	−5.136	−5.2435
**2**	−1.84	0.224	−1.349	−1.542	−2.426	−1.33475
**3**	−2.022	−3.229	−3.733	−6.686	−4.905	−4.4435
**4**	−2.248	−2.809	−5.465	−6.542	−5.7	−4.85575
**5**	−0.713	−2.454	−4.982	−6.283	−4.791	−4.37025
**6**	−1.224	−3.204	−4.899	−3.614	−4.753	−3.87825
**7**	−2.34	−4.861	−5.131	−6.065	−4.906	−4.6835
**8**	−2.186	−4.219	−4.642	−5.492	−5.491	−4.46425
**9**	−2.161	−2.982	−6.423	−4.796	−4.752	−4.60575
**10**	−2.539	−3.026	−4.264	−2.592	−5.942	−3.844
**11**	−3.154	−4.451	−3.228	−6.624	−5.506	−4.59925
**12**	−2.629	−3.893	−3.736	−7.32	−5.98	−4.7125
**13**	−3.102	−4.737	−4.357	−7.435	−5.599	−5.08225
**14**	−2.954	−4.292	−4.326	−6.493	4.61	−4.622

**Table 2:** Interactions between three top-scoring ligands and receptor for each protein, showing the site of interactions and the different types.

1SVC (NF-κB C62A)			Modified 1SVC (NF-κB)			4QTB (ERK1)			2ZOQ (ERK1)			2AZ5 (TNF-α)		
Ligand 1			Ligand 1			Ligand 9			Ligand 13			Ligand 12		
asn	139	H bond	His	144	H bond	met	125	H bond	met	125	aromatic H bond	tyr	151	aromatic H bond
gly	68	aromatic H bond	val	61	H bond	asp	123	aromatic H bond				tyr	59	aromatic H bond
			lys	149	H bond	tyr	53	H bond						
						lys	71	H bond						
Ligand 11			Ligand 7			Ligand 4			Ligand 1			Ligand 10		
lys	80	pi-cation	val	61	H bond	gln	122	aromatic H bond	met	125	aromatic H bond	tyr	59	pi pi stacking
			lys	149	H bond	asp	123	aromatic H bond				gly	121	aromatic H bond
						met	125	H bond				ser	60	aromatic H bond
Ligand 13			Ligand 13			Ligand 1			Ligand 12			Ligand 4		
lys	80	pi-cation	val	61	H bond	gln	122	H bond	met	125	aromatic H bond	leu	120	aromatic H bond
tyr	82	aromatic H bond	lys	149	H bond	asp	123	aromatic H bond	asp	184	H bond			
			glu	152	H bond	met	125	aromatic H bond						
						lys	131	H bond						

**Table 3:** Pharmaceutically relevant properties of screened derivatives provided with Qikprop. The scores for each property as well as the ideal range for drugs are provided.

Compound	Molecular Weight	H-Bond Donors	H-Bond Acceptors	logPo/w	Aqueous Solubility	CIQPlogS	logHERG	logBBB	Oral Absorption	Rule of 5 Deviations	Rule of 3 Deviations	logKhsa	PSA	Polrz	loPw	logKp	Ionization Potential	Electron Affinity
**1**	230.263	1	3.25	2.437	−3.129	−3.195	−4.187	−0.425	96.438	0	0	0.037	60.006	25.356	6.754	−2.349	9.251	0.89
**2**	230.263	1	3.25	2.457	−3.601	−3.195	−4.696	−0.632	93.825	0	0	0.088	59.703	26.256	6.907	−2.656	9.086	0.815
**3**	306.36	0	3	4.646	−5.154	−5.146	−5.793	−0.18	100	0	0	0.656	43.988	36.172	5.99	−0.867	9.261	1.045
**4**	325.366	0	5.75	3.089	−4.222	−4.215	−5.377	−0.871	96.99	0	0	0.049	79.072	36.465	7.974	−2.434	9.174	0.846
**5**	387.437	0	5.25	4.789	−6.306	−6.124	−6.85	−0.888	100	0	1	0.706	77.31	45.538	8.986	−1.606	9.154	1.1
**6**	388.425	0	6.75	3.574	−5.046	−5.45	−6.323	−1.184	95.484	0	1	0.221	89.717	43.848	10.248	−2.418	9.281	1.367
**7**	269.299	0	4.75	2.239	−3.952	−4.188	−4.218	−0.73	91.218	0	0	−0.18	70.8	28.925	6.456	−2.895	9.458	1.49
**8**	312.288	0	3.25	4.052	−4.836	−4.797	−4.377	0.159	100	0	0	0.369	45.14	30.794	4.683	−1.855	9.439	1.417
**9**	321.375	0	4.75	3.636	−4.334	−4.654	−5.148	−0.425	100	0	0	0.261	57.81	36.084	7.133	−1.77	9.286	1.201
**10**	320.387	0	3.25	4.694	−5.315	−5.328	−5.44	−0.171	100	0	0	0.702	44.835	36.982	5.736	−1.12	9.138	0.959
**11**	274.316	0	4	3.006	−3.391	−3.642	−4.207	−0.282	100	0	0	0.042	53.076	29.219	5.375	−1.955	9.104	1.046
**12**	314.38	1	4	3.656	−4.864	−4.65	−4.595	−0.629	100	0	0	0.541	65.288	34.705	7.326	−2.547	9.04	0.804
**13**	314.38	1	4	3.657	−4.895	−4.65	−4.638	−0.636	100	0	0	0.541	65.338	34.725	7.339	−2.546	9.038	0.803
**14**	274.316	0	4	3.325	−4.464	−3.642	−5.104	−0.381	100	0	0	0.19	53.034	31.23	5.502	−1.991	9.076	0.744
OS	244.29	0	3.25	3.121	−3.517	−3.353	−4.417	−0.085	100	0	0	0.111	45.124	27.869	4.983	−1.616	9.198	0.863
ideal range	0–500	0–5	0–10	−2–6.5	−6.5–0.5	−6.5–0.5	>−5	−3–1.2	70–100			−1.5–1.5	<120					

## Data Availability

All the data originating from this research are available from the authors under request.

## Supplementary Materials

The following supporting information can be downloaded at: https://www.mdpi.com/article/10.3390/life12040505/s1. Figure S1: Molecular modeling of highest-scoring compound in each binding site to demonstrate the precise location of binding on the protein, Figure S2: Molecular modeling of top-scoring compounds docked at binding site 1 detected by Schrodinger software in 1SVC (NF-κB C62A), Figure S3: Protein–ligand interactions of top-scoring compounds docked to binding site 1 1SVC (NF-κB C62A), Figure S4: Average docking scores for each protein screened and ligand screened, Figure S5: Docking score of each derivative with results of all the water conditions shown, Figure S6: Protein–ligand interaction diagrams of compounds docked to modified 1SVC (NF-κB), demonstrating all compounds that showed interactions with Cys62, Table S1: Scoring and residues of each potential binding site on 1SVC (NF-κB C62A) identified by Schrodinger Maestro software, Table S2: Residues included in each binding site detected by Maestro software, Table S3: Hydrogen bond interactions between water molecules and protein or screened ligands in binding site, Table S4: Residues included in each binding site, Table S5: Docking scores for each compound with each protein, showing every water condition.

## Limitations of the Study

The major limitation of the study is the lack of molecular dynamics of osthole derivates with selected targets.
