# Supplementary material for: Computational Screening of the Natural Product Osthole and Its Derivates for Anti-Inflammatory Activity
Source: Life (Basel). 2022 Mar 30;12(4):505. doi: 10.3390/life12040505 (PMC9030959; doi:10.3390/life12040505)
Supplement: Supplementary file 1 [file life-12-00505-s001.zip › life-1635558-supplementary.pdf]

life

MDPI

Article

Computational Screening of the Natural Product Osthole and Its Derivates for Anti-Inflammatory Activity

Angela Mosebarger [1], Rambabu N. Reddi [2], Ramkumar Menon [1] and Ananth Kumar Kammala [1,*]

[1] Division of Basic & Translational Research, Department of Obstetrics & Gynecology, The University of Texas Medical Branch at Galveston, 301 University Blvd., Galveston, TX 77555, USA; anmoseba@utmb.edu (A.M.); ra2menon@utmb.edu (R.M.)

[2] Department of Organic Chemistry, Weizmann Institute of Science, Rehovot, 234 Herzl St, PO Box 26 Rehovot 7610001 Israel; rambabu.reddi@weizmann.ac.il

* Correspondence: ankammal@utmb.edu; Tel.: +1-517-899-6963

Supplementary Materials

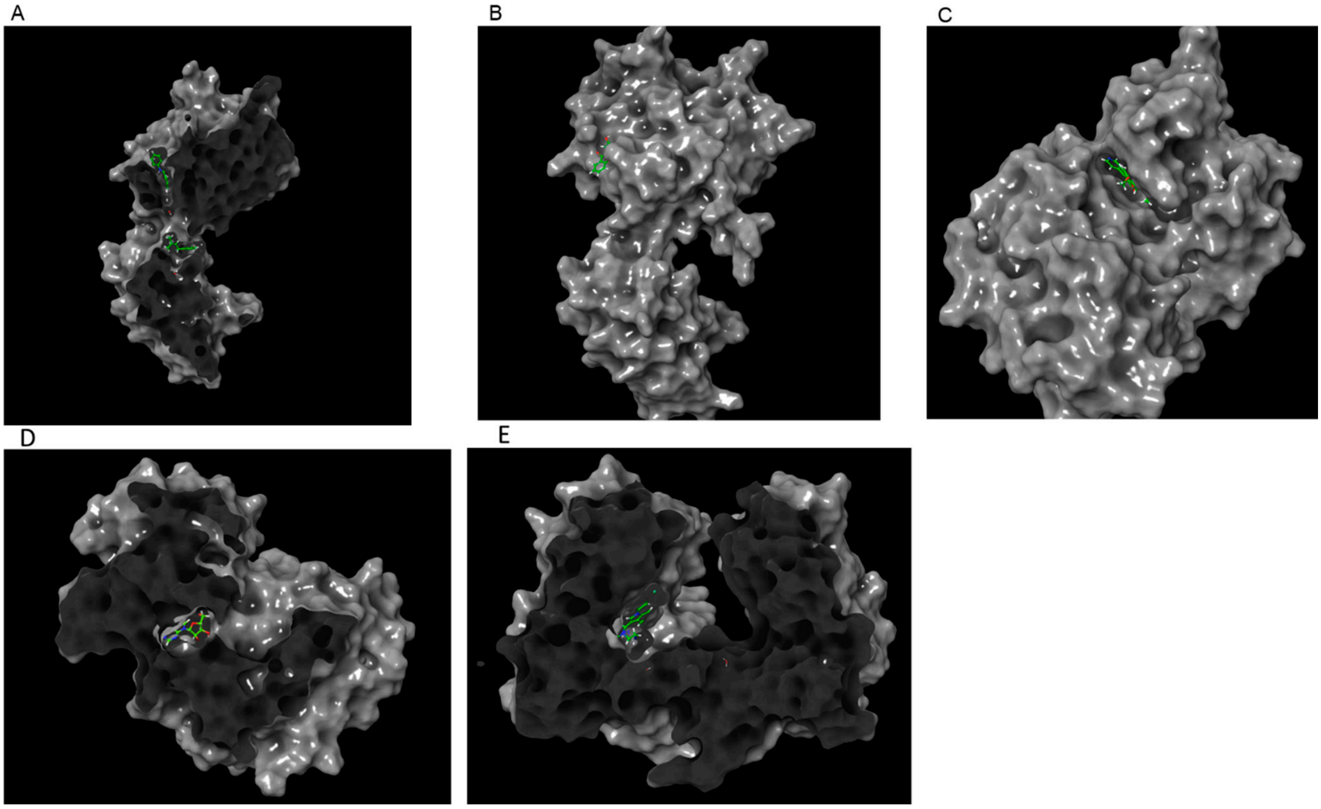

Figure S1. Molecular modeling of highest scoring compound in each binding site to demonstrate the precise location of binding on the protein. (A) 1SVC (NF-κB C62A) with inhibitor DHMEQ at Site 1 and Ligand 4 at Site 2 with water included in binding site; (B) Modified 1SVC (NF-kB) with DHMEQ docked at Cys62 with no water included in the binding site; (C). 4QTB (ERK1) with Ligand 9 with selected water molecules included in the binding site; (D) 2ZOQ (ERK1) with (2r,3r,4s,5r)-2-(4-amino-5-iodo-7h-pyrrolo[2,3-d]pyrimidin-7-yl)-5-(hydroxymethyl)tetrahydrofuran-3,4-diol with water included in the binding site; (E) 2AZ5 (TNF-α) with 6,7-dimethyl-3-[(methyl{2-[methyl({1-[3-(trifluoromethyl)phenyl]-1h-indol-3-yl}methyl)amino]ethyl}amino)methyl]-4h-chromen-4-one and water included in the binding site.

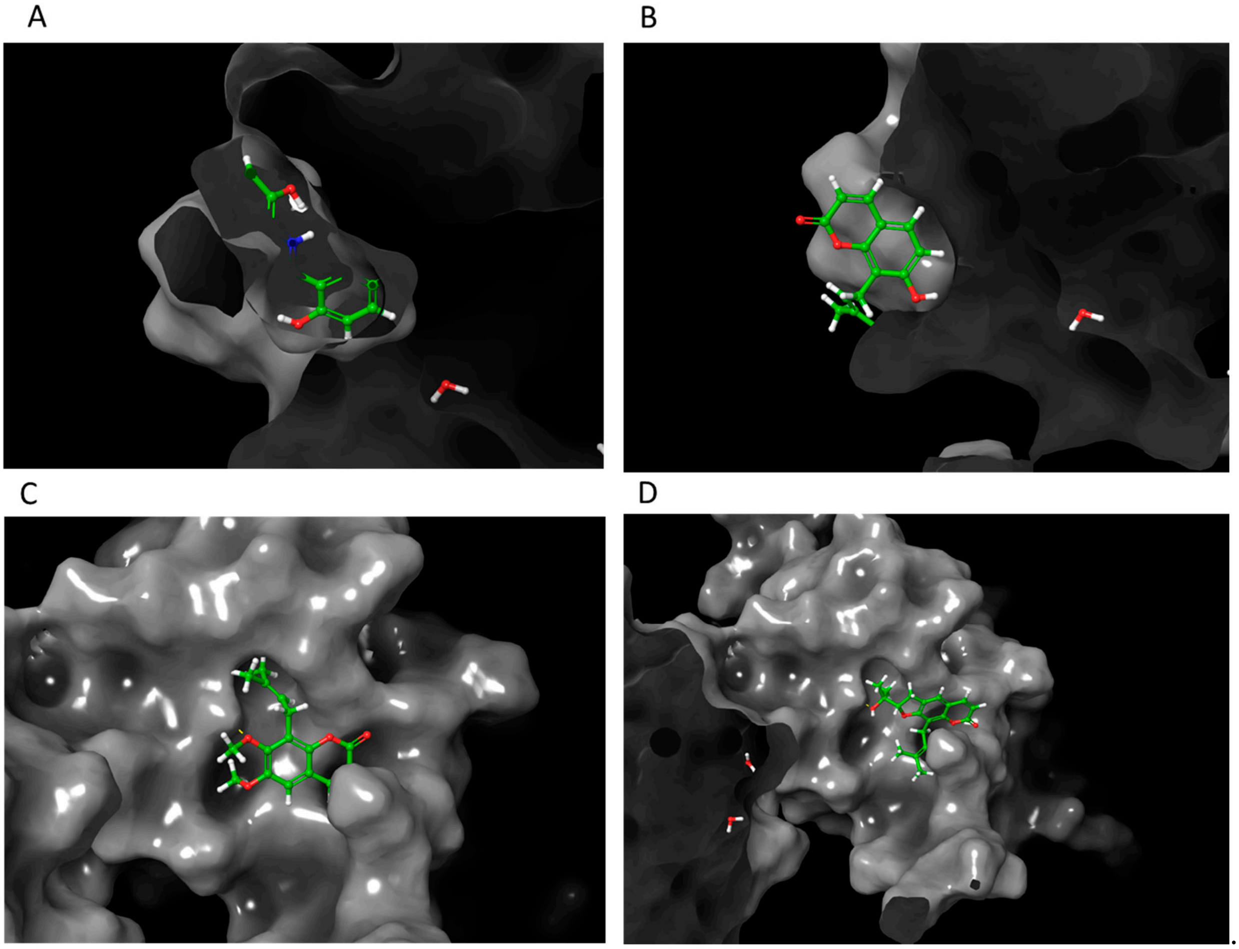

Figure S2. Molecular modeling of top-scoring compounds docked at binding site 1 detected by Schrodinger software in 1SVC (NF-κB C62A) with water included in the binding site. (A) Known inhibitor DHMEQ docked at Site 1; (B) Ligand 1 at binding site 1; (C) Ligand 11 at binding site 1; (D) Ligand 13 at binding site 1.

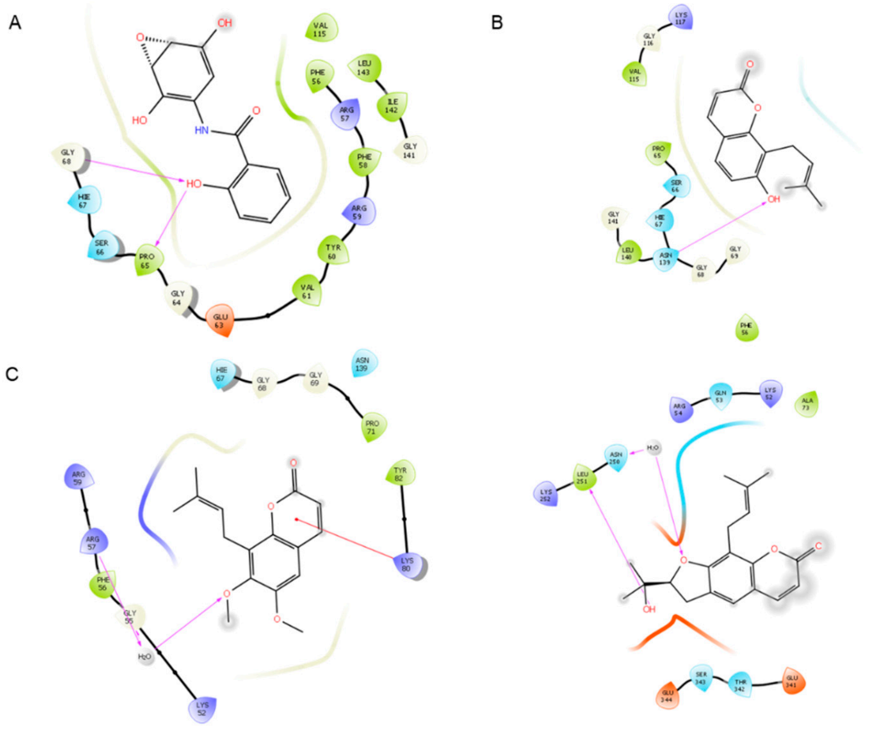

Figure S3. Scoring and residues of each potential binding site on 1SVC (NF-κB C62A) identified by Schrodinger Maestro software. Site 1 and site 2 were selected for analysis, although site 2 was later excluded due to low binding with the positive control DHMEQ.

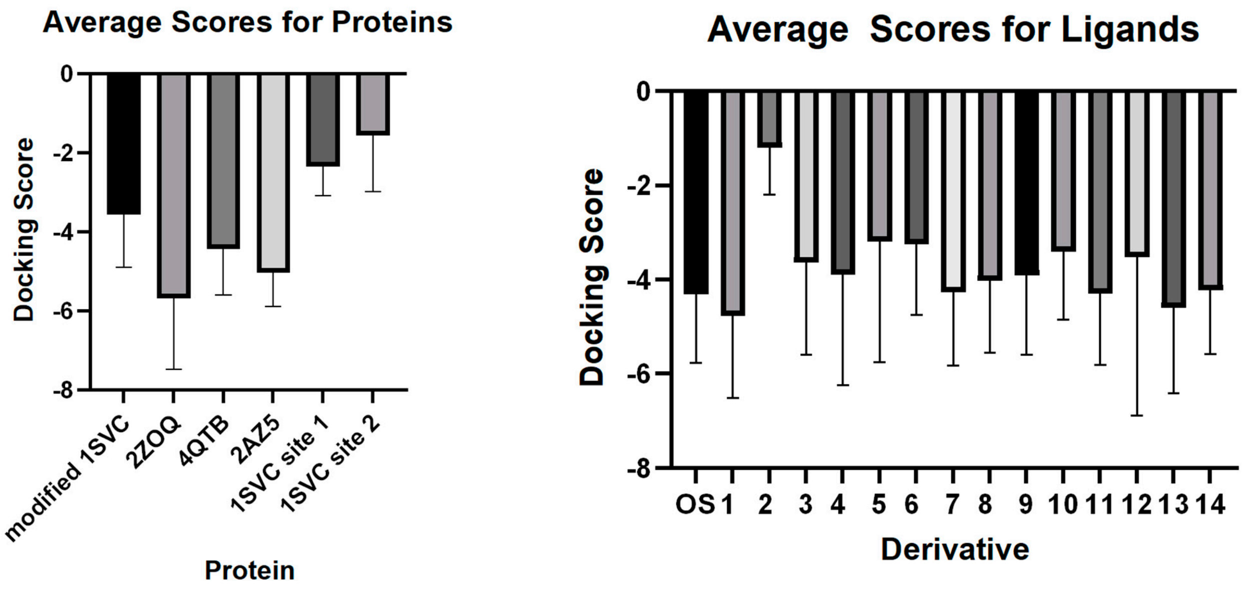

Figure S4. Average docking scores for each A. Protein screened and B. Ligand screened. Docking scores are given in kcal/mol. More negative scores correspond with better binding.

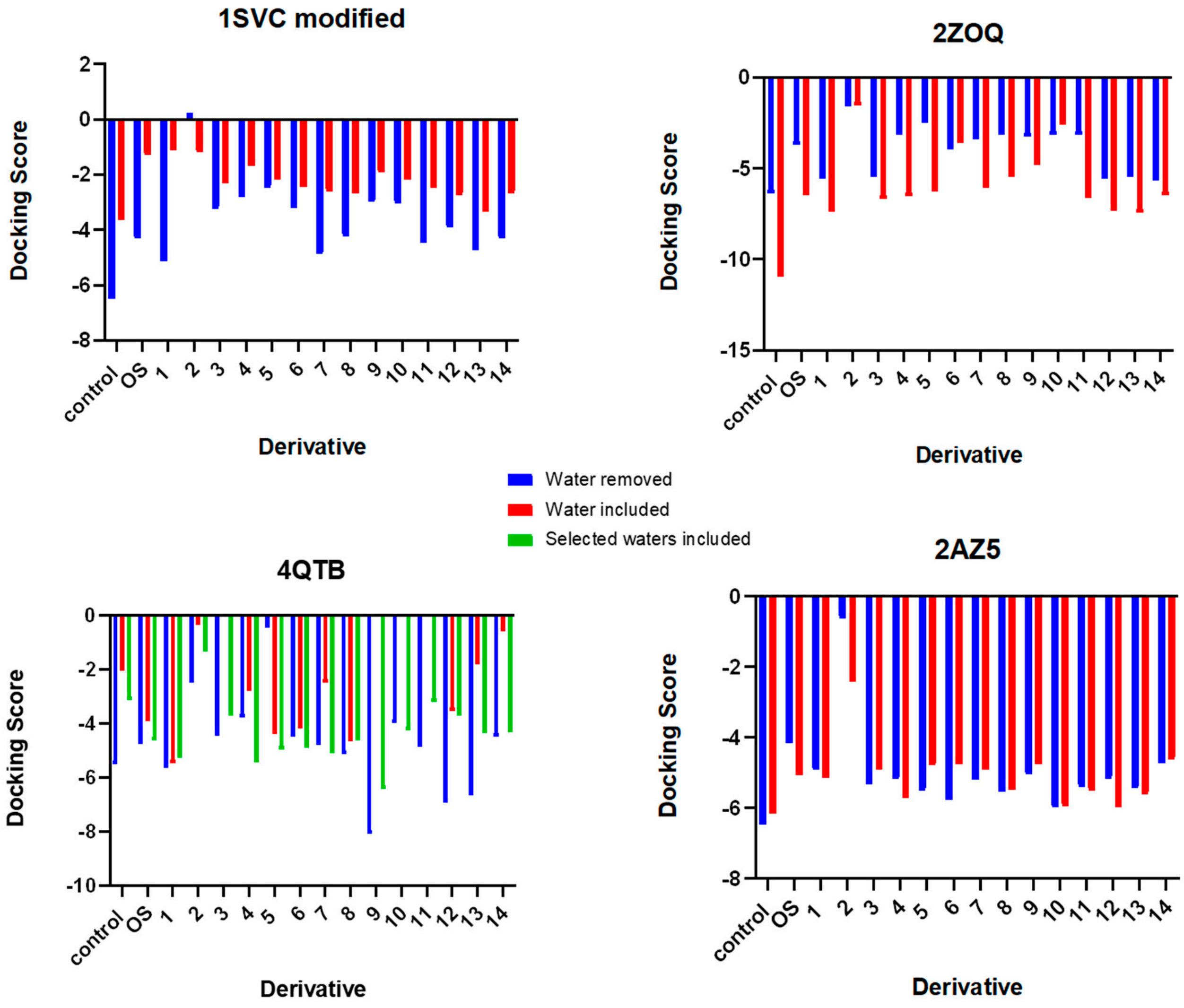

Figure S5. Docking score of each derivative with results of all the water conditions shown. Docking scores given in kcal/mol.

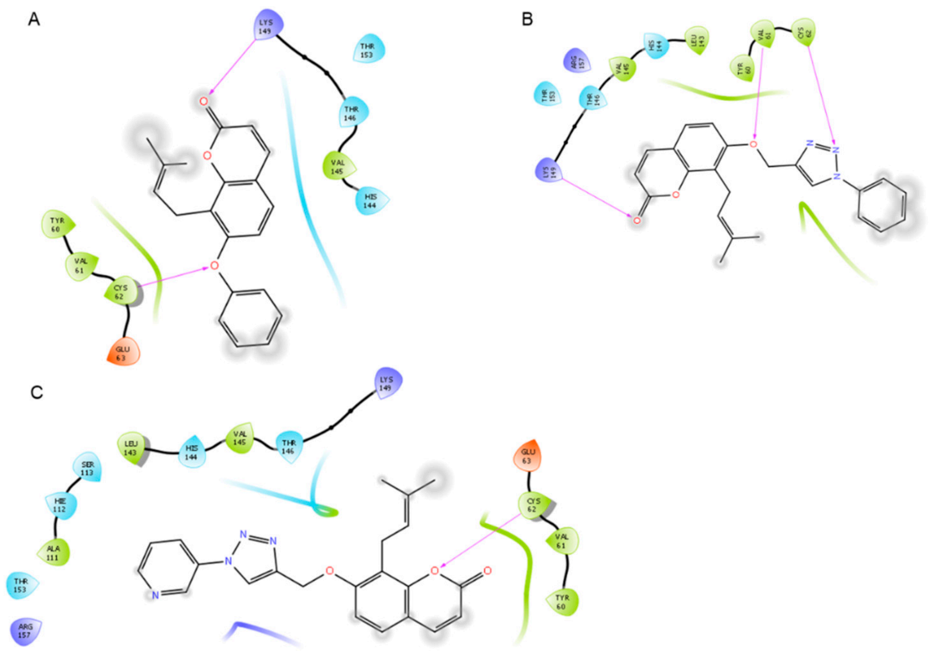

Figure S6. Protein-ligand interaction diagrams of compounds docked to modified 1SVC (NF-κB), demonstrating all compounds that showed interactions with Cys62. (A) Ligand 3; (B) Ligand 5; (C) Ligand 6.

Table S1. Scoring and residues of each potential binding site on 1SVC (NF-κB C62A) identified by Schrodinger Maestro software. Site 1 and site 2 were selected for analysis, although site 2 was later excluded due to low binding with the positive control DHMEQ.

1SVC binding site detection					
	Site score	Dscore	Volume	Balance	Residues
site1	0.935	0.963	154.007	0.938	Chain P: 56,57,58,59,60,61,64,65,67,68,69,71,80,82,115,116,117,139,141,142,143
site2	0.72	0.619	100.842	0.092	Chain P: 52,54,55,241,243,248,249,250,251,252,336,341,342,343,344
site3	0.714	0.57	65.513	0.241	Chain P: 102,103,104,105,106,108,110,204,207,208,209,212
site4	0.617	0.543	120.393	0.041	Chain P: 57,58,60,63,144,147,150,205,206,208,209,210,211,242,244,245,246
site5	0.612	0.55	47.334	0.417	Chain P: 116,117,118,124,126,136,137,138,139,140
ideal	>1	>1	>225	>0.3	

Table S2. Residues included in each binding site detected by Maestro software.

1SVC binding site detection									
**site 1**		**site 2**		**site 3**		**site 4**		**site 5**	
56	phe	52	lys	102	thr	57	arg	116	gly
57	arg	54	arg	103	asn	58	phe	117	lys
58	phe	55	gly	104	gly	60	tyr	118	his
59	arg	241	tyr	105	lys	63	glu	124	cys
60	tyr	243	ser	106	asn	144	his	126	val
61	val	248	ala	108	his	147	lys	136	gly
64	gly	249	ser	110	HIE	150	val	137	phe
65	pro	250	asn	204	gln	205	thr	138	ala
67	HIE	251	leu	207	glu	206	lys	139	asn
68	gly	252	lys	208	met	208	met	140	leu
69	gly	336	arg	209	asp	209	asp		
71	pro	341	glu	212	val	210	leu		
80	lys	342	thr			211	ser		
82	tyr	343	ser			242	asp		
115	val	344	glu			244	lys		
116	gly					245	ala		
117	lys					246	pro		
139	asn								
141	gly								
142	ile								
143	leu								

Table S3. Hydrogen bond interactions between water molecules and protein or screened ligands in binding site.

1SVC (NF-κB C62A)waters		modified 1SVC (NF-κB) waters		2ZOQ (ERK1) waters		4QTB (ERK1) waters		2AZ5 (TNF-α) waters	
**Residue**	**water molecule**	**Residue**	**water molecule**	**Residue**	**water molecule**	**Residue**	**water molecule**	**Residue**	**water molecule**
Arg54	HOH452	His144	HOH429	Asp128	HOH451	Asp184	HOH504	Gly121	HOH228
Gly55	HOH408	Glu63	HOH437		HOH543		HOH517		HOH252
Ser249	HOH452	Hie67	HOH437	Gly54	HOH391		HOH597		
Asn250	HOH423	Thr146	HOH430	Gln122	HOH389		HOH687		
Ile253	HOH405	ligand	HOH430	Glu126	HOH454	Ser74	HOH508		
Arg336	HOH452		HOH429	Ser170	HOH523		HOH662		
Glu344	HOH405		HOH420	Asp184	HOH479		HOH747		
				Ligand	HOH543	Arg84	HOH714		
					HOH391		HOH834		
					HOH523	Ala52	HOH727		
							HOH645		
						Asp128	HOH590		
							HOH1026		
						Ile48	HOH715		
						Gly51	HOH645		
						Tyr53	HOH508		
						Glu77	HOH662		
						Glu88	HOH517		
						Ile120	HOH553		
						Gln122	HOH553		
						Met125	HOH649		
						Lys131	HOH715		
						Ser170	HOH590		
						Asn171	HOH683		

Table S4. Residues included in each binding site. For the unmodified 1SVC, this was determined by the binding site detection tool in Maestro, for all other proteins, it was determined based on interactions with the known bound ligand.

1SVC (NF-κB C62A) binding site				modified 1SVC (NF-κB) binding site				4QTB (ERK1) binding site				2ZOQ (ERK1) binding site				2AZ5 (TNF-α) binding site			
amino acid	residue number	type of interaction	number of bonds	amino acid	residue number	type of interaction	number of bonds	amino acid	residue number	type of interaction	number of bonds	amino acid	residue number	type of interaction	number of bonds	amino acid	residue number	type of interaction	number of bonds
phe	56			tyr	60			Ile	48			ile	48			leu	55		
arg	57	aromatic H bond		val	61	H bond		ala	52			gly	49			leu	57		
phe	58			cys	62	H bond		tyr	53	pi cation		glu	50			tyr	59		
arg	59			leu	143			val	56			gly	51			ser	60		
tyr	60			his	144	H bond		ala	69			val	56			gln	61		
val	61			val	145			lys	71	H bond		ala	69			tyr	119		
gly	64			thr	146			ile	73			lys	71			leu	120	aromatic H bond	
pro	65	H bond		lys	149			pro	75			gln	122	halogen bond		gly	121		
hie	67			thr	153	H bond		tyr	81	pi-pi stacking		asp	123	H bond		gly	122		
gly	68	H bond and aromatic H bond	2					arg	84			leu	124			tyr	151	aromatic H bond	
val	115							thr	85			met	125	H bond and aromatic H bond	2	leu	157	salt bridge	
gly	141							glu	88	salt bridge		glu	126						
ile	142	aromatic H bond						ile	101			asp	128	H bond					
leu	143							gln	122	aromatic H bond		lys	131						
								asp	123	H bond		ser	170	H bond					
								leu	124			leu	173						
								met	125	H bond and aromatic H bond	2	cys	183						
								glu	126	aromatic H bond		asp	184	H bond					
								thr	127										
								asp	128	aromatic H bond									
								lys	131	H bond									
								leu	173										
								cys	183										
								asp	184	salt bridge									
								gly	186										

Table S5. Docking scores for each compound with each protein, showing every water condition. More negative binding scores correspond to better binding.

	Water removed						Water in binding site						Limited water	
	1SVC site1	1SVC site2	modified 1SVC	2ZOQ	4QTB	2AZ5	1SVC	1SVC site2	modified 1SVC	2ZOQ	4QTB	2AZ5	4QTB docking score	mean
control1	-6.365	-2.649	-6.46	-6.352	-5.533	-6.457	-4.561	-2.211	-3.635	-10.975	-2.076	-6.166	-3.151	-5.12238
OS	-2.597	-2.461	-4.294	-3.677	-4.777	-4.151	-2.837	-2.611	-1.28	-6.49	-3.944	-5.057	-4.627	-3.75408
1	-2.978	-2.765	-5.102	-5.553	-5.662	-4.918	-3.381	-2.358	-1.118	-7.375	-5.495	-5.136	-5.3	-4.39546
2	-1.664	-0.422	0.224	-1.579	-2.497	-0.608	-1.84	-0.26	-1.184	-1.542	-0.375	-2.426	-1.349	-1.194
3	-2.708	-0.548	-3.229	-5.439	-4.484	-5.333	-2.022	-1.294	-2.307	-6.686	0	-4.905	-3.733	-3.28369
4	-2.328	-2.557	-2.809	-3.133	-3.802	-5.168	-2.248	-0.642	-1.661	-6.542	-2.817	-5.7	-5.465	-3.45169
5	-0.305	-1.748	-2.454	-2.471	-0.462	-5.503	-0.713	0.078	-2.18	-6.283	-4.398	-4.791	-4.982	-2.78554
6	-1.064	-1.908	-3.204	-3.932	-4.521	-5.776	-1.224	-1.866	-2.42	-3.614	-4.206	-4.753	-4.899	-3.33746
7	-2.172	-2.173	-4.861	-3.394	-4.812	-5.198	-2.34	-2.365	-2.599	-6.065	-2.525	-4.906	-5.131	-3.73392
8	-2.424	-1.826	-4.219	-3.168	-5.158	-5.537	-2.186	-2.133	-2.682	-5.492	-4.668	-5.491	-4.642	-3.81738
9	-2.689	-2.149	-2.982	-3.233	-8.097	-5.043	-2.161	-2.335	-1.902	-4.796	0	-4.752	-6.423	-3.58169
10	-2.257	-0.859	-3.026	-3.172	-3.995	-5.981	-2.539	-2.124	-2.174	-2.592	0	-5.942	-4.264	-2.99423
11	-2.69	-2.72	-4.451	-3.137	-4.878	-5.391	-3.154	-2.85	-2.473	-6.624	0	-5.506	-3.228	-3.62323
12	-3.722	-1.146	-3.893	-5.578	-6.955	-5.165	-2.629	2.365	-2.722	-7.32	-3.552	-5.98	-3.736	-3.84869
13	-2.748	-2.227	-4.737	-5.49	-6.682	-5.435	-3.102	-2.342	-3.334	-7.435	-1.848	-5.599	-4.357	-4.25662
14	-2.946	-2.342	-4.292	-5.659	-4.499	-4.715	-2.954	-2.699	-2.658	-6.493	-0.624	-4.61	-4.326	-3.75515
